# State-of-The-Art and Applications of 3D Imaging Sensors in Industry, Cultural Heritage, Medicine, and Criminal Investigation

**DOI:** 10.3390/s90100568

**Published:** 2009-01-20

**Authors:** Giovanna Sansoni, Marco Trebeschi, Franco Docchio

**Affiliations:** Laboratory of Optoelectronics, University of Brescia / via Branze 38, Brescia I-25123, Italy; E-Mails: marco.trebeschi@ing.unibs.it; franco.docchio@ing.unibs.it

**Keywords:** 3D sensors, surface quality control, reverse engineering, optical triangulation, crime scene investigation, heritage restoration, virtual reality

## Abstract

3D imaging sensors for the acquisition of three dimensional (3D) shapes have created, in recent years, a considerable degree of interest for a number of applications. The miniaturization and integration of the optical and electronic components used to build them have played a crucial role in the achievement of compactness, robustness and flexibility of the sensors. Today, several 3D sensors are available on the market, even in combination with other sensors in a “sensor fusion” approach. An importance equal to that of physical miniaturization has the portability of the measurements, via suitable interfaces, into software environments designed for their elaboration, e.g., CAD-CAM systems, virtual renders, and rapid prototyping tools. In this paper, following an overview of the state-of-art of 3D imaging sensors, a number of significant examples of their use are presented, with particular reference to industry, heritage, medicine, and criminal investigation applications.

## 1. Introduction

In recent years, demand for optical 3D imaging sensors has become increasingly relevant, and has led to the development of instruments that are now commercially available [1]. During the 1970s and 1980s their development was mainly performed in research laboratories, and was aimed at designing new techniques exploiting the use of light beams (both coherent and incoherent) instead of contact probes, in view of their application in the mechanical manufacturing industry, for measurement and quality control applications. Novel measurement principles were proposed, and suitable prototypes were developed and characterized to prove their performances [2–9].

In parallel, we participated in a considerable effort directed towards the miniaturization and integration of optical light sources, detectors and components, in the electronic equipment and the mechanical structure of the sensors. In the last decade, the availability of techniques and components has led to the production of a wide spectrum of commercially available devices, with measurement resolution from a few nanometers to fractions of a meter, and ranges from microns to a few kilometers. In recent times, the trend has been to produce devices at decreased costs and with increased ruggedness and portability.

The problem of manipulating, editing, and storing the measured data was approached at the software level, by developing a very powerful suite of programs that import and manipulate the data files, and output them in popular and well-standardized formats, such as the DXF and IGES formats for CAD applications, the STL format for rapid prototyping machines, and the VRML and 3D formats for visualization.

As a result, the interest in the use of 3D imaging sensors has increased. In the mechanical and manufacturing industry, surface quality control, microprofiling and macroprofiling are often carried out on a contactless, optical basis. Optical probes and contact probes are used in combination: this occurs whenever both the accuracy of the measurement and the efficiency of the process are the critical points [10]. It is significant that, today, one of the input requirements in the design of Coordinate Measuring Machines (CMMs) is represented by the possibility of mounting optical 3D measurement sensors *and* contact probes.

In addition, 3D imaging sensors are becoming of interest in combination with two dimensional (2D) vision sensors, especially in robotic applications, for collision avoidance, and to solve ‘removing-from-heap’ and assembling problems [11]. As a result, those companies traditionally focused on the development of 2D vision systems, are now enlarging their product spectrum by including 3D range sensors.

The use of optical depth sensors has gone beyond the mechanical field for which they were originally intended. Examples of application fields are geology, civil engineering, archaeology, reverse engineering, medicine, and virtual reality [12–16].

The aim of this paper is to briefly review the state of the art concerning 3D sensing techniques and devices, and to present their applications in industry, cultural heritage, medicine and forensics. It focuses in part on the research carried out at our Laboratory, and briefly describes related approaches developed by other groups.

The paper has the following structure: Section 2 overviews 3D imaging techniques, and evaluates the different approaches to highlight which method can be used and what are the main applications. Section 3 gives an insight of the results achieved in the mentioned applications. As far as the industrial field is concerned, the topics of surface quality control, dimensional measurement and reverse engineering are covered. Significant results in the cultural heritage field, both achieved by us and by other research groups are shown. The approaches developed in the medical field are briefly reviewed, and the *post mortem* analysis of lesions is cited as an example of the latest applications in this area. The last section is dedicated to the use of 3D imaging techniques for the accurate, fast and non-invasive measurement and modeling of crime scenes.

## 2. Overview of 3D imaging techniques

3D imaging sensors generally operate by projecting (in the active form) or acquiring (in the passive form) electromagnetic energy onto/from an object followed by recording the transmitted or reflected energy. The most important example of transmission gauging is the industrial computer tomography (CT), which uses high-energy x-rays and measures the radiation transmitted through the object.

Reflection sensors for shape acquisition can be subdivided into non-optical and optical sensing. Non-optical sensing includes acoustic sensors (ultrasonic, seismic), electromagnetic (infrared, ultraviolet, microwave radar, etc…) and others. These techniques typically measure distances to objects by measuring the time required for a pulse of sound or microwave energy to bounce back from an object.

In reflection optical sensing, light carries the measurement information. There is a remarkable variety of 3D optical techniques, and their classification is not unique. In this section, we mention the more prominent approaches, and we classify them as shown in Table 1.

The 3D techniques are based on optical triangulation, on time delay, and on the use of monocular images. They are classified into passive and active methods. In passive methods, the reflectance of the object and the illumination of the scene are used to derive the shape information: no active device is necessary. In the active form, suitable light sources are used as the internal vector of information. A distinction is also made between direct and indirect measurements. Direct techniques result in range data, i.e., into a set of distances between the unknown surface and the range sensor. Indirect measurements are inferred from monocular images and from prior knowledge of the target properties. They result either in range data or in surface orientation.

Excellent reviews of optical methods and range sensors for 3D measurement are presented in references [1–7]. In [1] a review of 20 years of development in the field of 3D laser imaging with emphasis on commercial systems available in 2004 is given. Comprehensive summaries of earlier techniques and systems are provided in the other publications. A survey of 3D imaging techniques is also given in [8] and in [9], in the wider context of both contact and contactless 3D measurement techniques for the reverse engineering of shapes.

### 2.1 Laser triangulators

**Figure 1.**
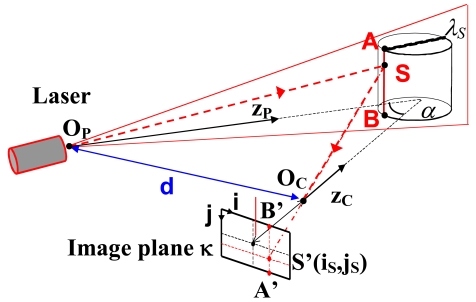
Schematics of the triangulation principle.

Both single-point triangulators and laser stripes belong to this category. They are all based on the active triangulation principle [17]. Figure 1 shows a typical system configuration. Points O_P_ and O_C_ are the exit and the entrance pupils of a laser source and of a camera. Their mutual distance is the baseline d. The optical axes z_P_ and z_C_ of the laser and the camera form an angle of α degrees.

The laser source generates a narrow beam, impinging the object at point S (single-point triangulators). The back scattered beam is imaged at point S′ at image plane κ. The measurement of the location (i_S_, j_S_) of image point S′ defines the line of sight $\bar{{\text{S}}^{\prime }\;{\text{O}}_{\text{C}}}$, and, by means of simple geometry, yields the position of S. The measurement of the surface is achieved by scanning. In a conventional triangulation configuration, a compromise is necessary between the Field of View (FOV), the measurement resolution and uncertainty, and the shadow effects due to large values of angle α.

To overcome this limitation, a method called ‘synchronized scanning’ has been proposed. Using the approach illustrated in [18, 19], a large field of view can be achieved with a small angle α, without sacrificing range measurement precision.

Laser stripes exploit the optical triangulation principle shown in Figure 1. However, in this case, the laser is equipped with a cylindrical lens, which expands the light beam along one direction. Hence, a plane of light is generated, and multiple points of the object are illuminated at the same time. In the figure, the light plane is denoted by λ_S_, and the illuminated points belong to the intersection between the plane and the unknown object (line $\bar{\text{AB}}$). The measurement of the location of all the image points from A′ to B′ at plane κ allows the determination of the 3D shape of the object in correspondence with the illuminated points. For dense reconstruction, the plane of light must scan the scene [20–22]. An interesting enhancement of the basic principle exploits the Biris principle [23]. A laser source produces two stripes by passing through the Bi-Iris component (a mask with two apertures). The measurement of the point location at the image plane can be carried out on a differential basis, and shows decreased dependence on the object reflectivity and on the laser speckles.

One of the most significant advantages of laser triangulators is their accuracy, and their relative insensitivity to illumination conditions and surface texture effects. Single-point laser triangulators are widely used in the industrial field, for the measurement of distances, diameters, thicknesses, as well as in surface quality control applications. Laser stripes are becoming popular for quality control, reverse engineering, and modeling of heritage. Examples are the Vivid 910 system (Konica Minolta, Inc.), the sensors of the SmartRay series (SmartRay GmbH, Germany) and the ShapeGrabber systems (ShapeGrabber Inc., CA, USA). 3D sensors are designed for integration with robot arms, to implement “pick and place” operations in de-palletizing stations. An example is the Ranger system (Sick, Inc.). The NextEngine system (NextEngine, Inc., CA, USA) and the TriAngles scanner (ComTronics, Inc., USA) device deserve particular attention, since they show how the field is progressing. These two systems, in fact, break the price barrier of laser-based systems, which is a limiting factor in their diffusion.

### 2.2 Structured light

**Figure 2.**
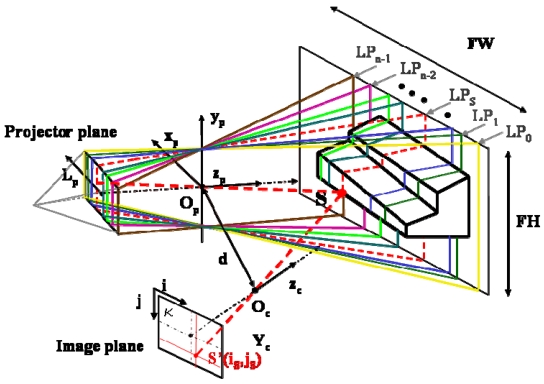
Principle of measurement in structured light systems.

Structured light based sensors share the active triangulation approach above mentioned. However, instead of scanning the surface, they project bi-dimensional patterns of non-coherent light, and elaborate them to obtain the range information for each viewed point simultaneously. A single pattern as well as multiple patterns can be projected. In both cases, the single light plane λ_S_ shown in Figure 1 is replaced by a bundle of geometrical planes, schematically shown in Figure 2. The planes are indexed along the LP coordinate at the projector plane. The depth information at object point S is obtained as the intersection between line sight SS′ and the plane indexed by LP=LP_S_ [25]. The critical point is to guarantee that different object points are assigned to different indexes along the LP coordinate. To this aim, a large number of projection strategies have been developed. The projection of grid patterns [26, 27], of dot patterns [28], of multiple vertical slits [29] and of multi-color projection patterns [30] have been extensively studied. Particular research has been carried out on the projection of fringe patterns [31, 32], that are particularly suitable to maximize the measurement resolution.

**Figure 3.**
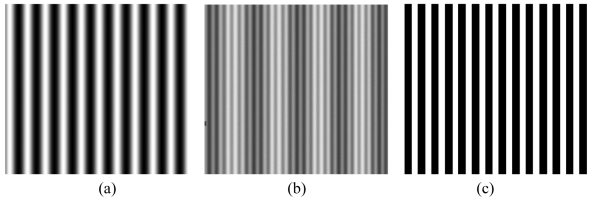
Example of fringe projection schemes. (a) Fringe pattern of sinusoidal fringes. (b) Superposition of two sinusoidal fringe patterns at different frequencies. (c) Projection of fringes of rectangular profile.

As an example, in Figure 3, three fringe patterns are shown. The first one is formed by fringes of sinusoidal profile, the second one is obtained by using the superposition of two patterns with sinusoidal fringes at different frequencies [33], and the last one is formed by fringes of rectangular profile [34].

Recent trends in the development of structured light systems are aimed at increasing the speed of projection of multiple patterns, in order to allow the real-time acquisition of the surfaces, especially for motion planning and human body acquisitions. The MobilCam 3D system (ViaLUX GmbH, Germany), and the Broadway scanner (Artec Group, Inc., CA, USA) belong to this category.

### 2.3 Stereo vision

The stereo vision method represents the passive version of structured light techniques. Here, two (or more) cameras concurrently capture the same scene. In principle, the reconstruction by stereo approach uses the following sequence of processing steps: (i) image acquisition, (ii) camera modeling, (iii) feature extractions, (iv) correspondence analysis and (v), triangulation. No further equipment (e.g. specific light sources) and no special projections are required. The remarkable advantages of the stereo approach are the simplicity and the low cost; the major problem is the identification of common points within the image pairs, i.e., the solution of the well-known correspondence problem. Moreover, the quality of the shape extraction depends on the sharpness of the surface texture (affected by variations in surface reflectance). Stereo vision has significant applications in robotics and in computer vision, where the essence of the problem is not the accurate acquisition of highly quality data, but, rather, their interpretation (for example for motion planning, collision avoidance and grasping applications).

A variant of the stereo vision approach implies the use of multiple images from a single camera, which captures the object in a sequence of images from different views (shape from video). A basic requirement for this technique is that the scene does not contain moving parts. 3D urban modeling and object detection are typical applications [35–38].

Stereo vision based on the detection of the object silhouette has recently become very popular. The Stereo approach is exploited, but the 3D geometry of the object is retrieved by using the object contours under different viewing directions. One of the most prominent applications of this method is the modeling of 3D shapes for heritage, especially when the texture information is captured together with the range [39]. Among the devices on the market, the PrOMT.stereo system (SOLVing3D GmbH, Germany) shows high performance and simplicity of use.

### 2.4 Photogrammetry

Photogrammetry obtains reliable 3D models by means of photographs. In digital photogrammetry digital images are used. The elaboration pipeline consists basically of the following steps: camera calibration and orientation, image point measurements, 3D point cloud generation, surface generation and texture mapping. Camera calibration is crucial in view of obtaining accurate models. Image measurement can be performed by means of automatic or semi-automatic procedures. In the first case, very dense point clouds are obtained, even if at the expense of inaccuracy and missing parts. In the second case, very accurate measurements are obtained, over a significantly smaller number of points, and at increased elaboration and operator times [40–42]. Typical applications of photogrammetry, besides the aerial photogrammetry, are in close range photogrammetry, for city modeling, medical imaging and heritage [48–43].

Reliable packages are now commercially available for complete scene modeling or sensor calibration and orientation. Examples are the Australis software (Photometrics, Australia), Photomodeler (Eos Systems, Inc., Canada), Leica Photogrammetry Suite (Leica Geosystems GIS & Mapping), and Menci (Menci Software srl, Italy).

Both stereo vision and photogrammetry share the objective of obtaining 3D models from images and are currently used in computer vision applications. The methods rely on the use of camera calibration techniques. The elaboration chain is basically the same, even if linear models for camera calibration and simplified point measurement procedures are used in computer vision applications. In fact, in computer vision the automation level of the overall process is more important with respect to the accuracy of the models [44].

In recent times, the photogrammetric and computer vision communities have proposed combined procedures, for increasing both the measurement performance and the automation levels. Examples are in the use of laser scanning and photogrammetry [45–47] and in the use of stereo vision and photogrammetry for facade reconstruction and object tracking [48, 50]. On the market, combinations of structured light projection with photogrammetry are available (Atos II scanner, Gom GmbH, Germany) for improved measurement performance.

### 2.5 Time of Flight

Surface range measurement can be made directly using the radar time-of-flight principle. The emitter unit generates a laser pulse, which impinges onto the target surface. A receiver detects the reflected pulse, and suitable electronics measures the roundtrip travel time of the returning signal and its intensity. Single point sensors perform point-to-point distance measurement, and scanning devices are combined to the optical head to cover large bi-dimensional scenes. Large range sensors allow measurement ranges from 15 m to 100 m (reflective markers must be put on the target surfaces). Medium range sensors can acquire 3D data in shorter ranges. The measurement resolutions vary with the range. For large measuring ranges, time-of flight sensors give excellent results. On the other side, for smaller objects, about one meter in size, attaining 1 part per 1,000 accuracy with time-of-flight radar requires very high speed timing circuitry, because the time differences are in the pico-second range. A few amplitude and frequency modulated radars have shown promise for close range distance measurement [37, 51, 52].

In many applications, the technique is range-limited by allowable power levels of laser radiation, determined by laser safety considerations. Additionally, time-of-flight sensors face difficulties with shiny surfaces, which reflect little back-scattered light energy except when oriented perpendicularly to the line of sight.

### 2.6 Interferometry

Interferometric methods operate by projecting a spatially or temporally varying periodic pattern onto a surface, followed by mixing the reflected light with a reference pattern. The reference pattern demodulates the signal to reveal the variation in surface geometry. The measurement resolution is very high, since it is a fraction of the wavelength of the laser radiation. For this reason, surface quality control and microprofilometry are the most explored applications. Use of multiple wavelengths and of double heterodyne detection lengthens the non-ambiguity range. Systems based on this principle are successfully used to calibrate CMMs. Holographic interferometry relies on mixing coherent illumination with different wave vectors. Holographic methods typically yield range accuracy of a fraction of the light wavelength over microscopic fields of view [5, 53].

### 2.7 Moiré fringe range contours

Moiré fringe range contours are obtained by illuminating the scene with a light source passing through a grating, and viewing the scene with a laterally displaced camera through an identical second grating. The resulting interference pattern shows equidistant contour lines, but there is no information about the corresponding depth values. In addition, it is impossible to have direct information about the sign of the concavities. Moirè methods can have phase discrimination problems when the surface does not exhibit smooth shape variations [54].

### 2.8 Shape from focusing

Depth and range can be determined by exploiting the focal properties of a lens. A camera lens can be used as a range finder by exploiting the “depth of view” phenomena. In fact, the target image is blurred by an amount proportional to the distance between points on the object and the in-focus object plane. This technique has evolved from the passive approach to an active sensing strategy. In the passive case, surface texture is used to determine the amount of blurring. Thus, the object must have surface texture covering the whole surface in order to extract shape. The active version of the method operates by projecting light onto the object to avoid difficulties in discerning surface texture. Most prior work in active depth from focus has yielded moderate accuracy up to one part per 400 over the field of view [55]. There is a direct trade-of between depth of view and field of view; satisfactory depth resolution is achieved at the expense of a sub-sampling the scene, which in turn requires some form of mechanical scanning to acquire range measurement over the whole scene. Moreover, spatial resolution is non-uniform. Specifically, depth resolution is substantially less than resolution perpendicular to the observation axis. Finally, objects not aligned perpendicularly to the optical axis and having a depth dimension greater than the depth of view will come in to focus at different ranges, complicating the scene analysis and interpretation [56].

### 2.9 Shape from shadows

This technique is a variant of the structured light approach. The 3D model of the unknown object is rebuilt by capturing the shadow of a known object projected onto the target when the light is moving. Low cost and simple hardware are the main advantages of this approach, even if at the expense of low accuracy [57].

### 2.10 Shape from texture

The idea is to find possible transformations of texture elements (texels) to reproduce the object surface orientation. For example, given a planar surface, the image of a circular texel is an ellipse with varying values of its axes depending on its orientation [58]. A review of the techniques developed in this area is in reference [37]. These methods yield simple, low cost hardware. However, the measurement quality is low.

### 2.11 Shape from shading

Shape-from-shading requires the acquisition of the object from one viewing angle and varying the position of the light source, which results in varying the shading on the target surface. A variant of this method implies the variation of the lighting conditions. A review of the algorithms able to extract the shape information, starting from the reflectance map of the image, is in reference [59]. The acquisition hardware is very simple, and low cost. However, low accuracy is obtained, especially in the presence of external factors influencing the object reflectance. The measurement software is rather complex.

### 2.12 Strengths and weaknesses of 3D imaging techniques

The main characteristics of optical range imaging techniques are summarized in Table 2. The comments on the table are quite general in nature, and a number of exceptions are known. Strengths and weaknesses of the different techniques are strongly application-dependent. The common attribute of being non-contact is an important consideration in those applications characterized by either fragile, deformable objects, or on-line measurements or hostile environments. Acquisition time is another important aspect, when the overall cost of the measurement process suggests the reduction of the operator time even at the expense of deteriorating the quality of the data. On the other hand, active vision systems using a laser beam that illuminates the object inherently involve safety considerations, and possible surface interaction effects. In addition, the speckle effect introduces disturbances that represent a serious limit to the accuracy of the measurements [60]. Fortunately, recent advances in LEDs technology yield a valuable solution to this problem, since they are suitable candidates as light projectors in active triangulation systems.

The dramatic evolution of CPUs and memories has led in the last three years to elaboration performances that were by far impossible before, at low costs. For this reasons, techniques that are computation demanding (e.g., passive stereo vision) are now more reliable and efficient.

The selection of which sensor type should be used to solve a given depth measurement problem is a very complex task, that must consider (i) the measurement time, (ii) the budget, and (iii) the quality expected from the measurement. In this respect, 3D imaging sensors may be affected by missing or bad quality data. Reasons are related to the optical geometry of the system, the type of projector and/or acquisition optical device, the measurement technique and the characteristics of the target objects. The sensor performance may depend on the dimension, the shape, the texture, the temperature and the accessibility of the object. Relevant factors that influence the choice are also the ruggedness, the portability, and the adaptiveness of the sensor to the measurement problem, the easiness of the data handling, and the simplicity of use of the sensor.

### 2.13 The software for the elaboration of 3D data

Besides the acquisition of 3d raw data, the construction of 3D models requires the following steps:

1. Registration of multiple images;
2. Integration of registered images into a fused single point cloud and determination of the tessel mesh that models the surface;
3. Acquisition of reflection data, for surface visualization.

The registration step consists of the alignment in a common reference frame of all the views captured to cover the whole surface. A relevant number of techniques have been proposed. ICP based algorithms, for example, allow the pair wise registration of adjacent views [61, 62]. Multi-view registration methods carry out the alignment of multiple views following a global approach [63].

The integration step has been widely studied by the computer graphics community, to develop efficient methods for the creation of meshes. Volumetric methods [64, 65], mesh stitching [66, 67], region growing techniques [68, 69], and sculpting based methods [70] are of outmost importance in this field.

The acquisition of reflectance data is required for the correct visualization of the 3D models. Typical applications are for constructing 3D models for virtual reality, animation, and cultural heritage. The research developed in this area has led to approaches based either on parametric reflectance models or on a set of colour images of the object [71, 72]. Excellent reviews of these methods are in references [73–75].

There are a number of commercial packages available for manipulation of 3D data. Examples are PolyWorks (InnovMetric, Inc., Canada), Geomagic (Raindrop Geomagic, Inc., USA), Rapidform (Inus Technology, Inc. & Rapidform, Inc.), Rinhoceros (Rino3D Inc., USA), Surfacer (Powershape, Inc., UK). They provide view importing, editing, alignment and fusion. Specific modules allow mesh-editing, compression, rendering, visualizing and inspecting. Some of them provide the tools for the construction of the CAD model from the mesh. It is worth noting that in general, the operations are performed in a semi-automatic way, i.e., the operator skill is crucial to optimize the various steps, and to keep under control the influence of the alignment errors and of the editing operations over the 3D models.

## 3. Experience in the application of 3D imaging sensors

A wide range of applications is possible nowadays, thanks to the availability of engineered sensors, of laboratory prototypes, and of suitable software environments to elaborate the measurements. This section presents a brief insight of the most important fields where 3D imaging sensors can be fruitfully used. For each one, an application example that has been carried out in our Laboratory is presented.

### 3.1 Industrial applications

Typical measurement problems in the industrial field deal with (i) the control of machined surfaces, for the quantitative measure of roughness, waviness and form factor, (ii) the dimensional measurement and the quality control of products, and (iii) the reverse engineering of complex shapes.

#### 3.1.1 Surface quality control

Microprofilometry techniques and sensors are the best suited to carry out the measurement processes in the surface control field. In this application, the objective is to gauge the 3D quantitative representation of the surfaces in measurement intervals from a few microns to tens of millimeters, with resolutions from a few tens of nanometers to tens of microns. A typical application is the measurement of roughness and of waviness and the gauging of the 3D topology of machined surfaces. Typical contactless probes are represented by interferometers, autofocus and triangulator sensors. Since these are single-point devices, the measurement must be accomplished by scanning suitable samples of the surfaces. Techniques have been proposed by various authors. Laser integrated measurement of surface roughness and micro-displacement is proposed in [76]. In [77], a multi-detector triangulation laser probe is developed and mounted on a CMM for very accurate surface inspection.

**Figure 4.**
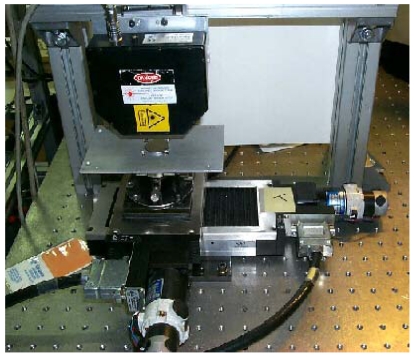
The microprofilometer setup developed at the laboratory for off-line microprofilometry applications.

Whenever the control is off-line, a system like that shown in Figure 4 can represent a good solution. Two automatic micropositioning high-accuracy slits carry out bi-dimensional x and y scanning of the surface under test. Depending on the measurement range and the resolution, an active autofocus sensor or a triangulator are mounted as the measuring probes along the z direction.

**Figure 5.**
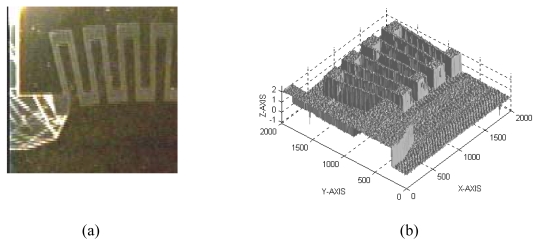
3D topography of a 2 mm×2 mm surface of a thin-film sensor. (a) Image of the measured surface. (b) 3D plot of the surface topography.

An example of the system performance is shown in Figure 5. The measured surface in Figure 5a is the 2 mm×2 mm portion of a thin-film sensor. The sampling step along the x and y coordinates is equal to 10 μm. Due to the extremely small height range of the target, an autofocus sensor (LNS 2.3/4.4 Dynavision, now LMI Inc) was selected as the measurement probe. The 3D topographic map of the surface is presented in Figure 5b. The measurement units are in μm.

**Figure 6.**
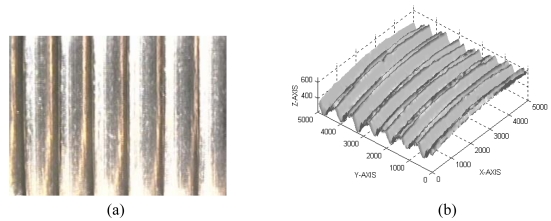
Example of the measurement by using the LTS 15/3 triangulator sensor. (a) The specimen. (b) The measured point cloud.

Another interesting example of the application of the setup in Figure 4 is the measurement of the turning machined surface shown in Figure 6a. In this case, a single-point triangulator (LTS 15/3, Dynavision) was used to capture the depth map shown in Figure 6b. Here, the dimension of the sample is equal to 5 mm × 5 mm, and the plotted range is from 0 to 600 μm.

**Figure 7.**
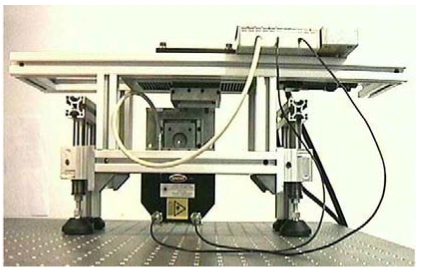
View of the mechanical frame that holds the sensor during industrial testing.

**Figure 8.**
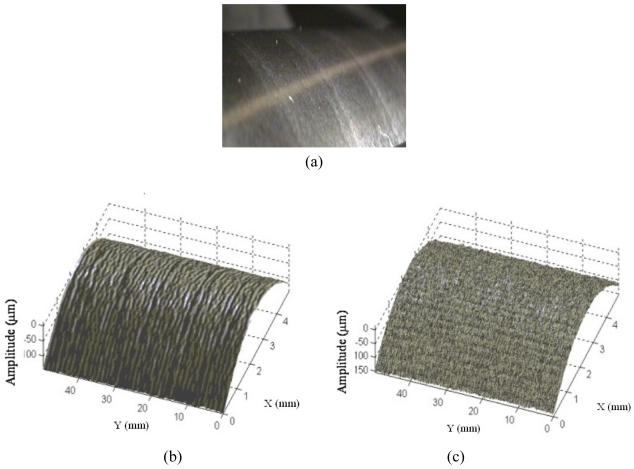
Example of 3D acquisition in the micro range. (a) The inspected surface. (b) 3D profile of the surface waviness. (c) 3D profile of the surface roughness.

For on-line measurements, our Laboratory has developed the sensor shown in Figure 7. The system works on typical grinding stations, used to recondition rolls for milling; it integrates a surface profiler and a light scattering unit in a compact setup suitable for industrial applications [81]. The optical profiler is the same triangulation sensor as in Figure 6, suitable for measuring the surface waviness. The scattering sensor is integrated on the triangulator for the detection of the surface roughness. Figure 8 shows a measurement example.

#### 3.1.2 Dimensional measurement and quality control

The dimensional measurement of complex surfaces is a really wide-range field of application, since it involves the 3D acquisition of shapes in the range from tens of millimetres to a few meters, with measurement resolution from one hundred microns to a few centimetres. The choice of the sensor depends on the object shape and dimension, on the environmental conditions, on the time available to perform the measurement, and, of course, on the measurement requirements.

**Figure 9.**
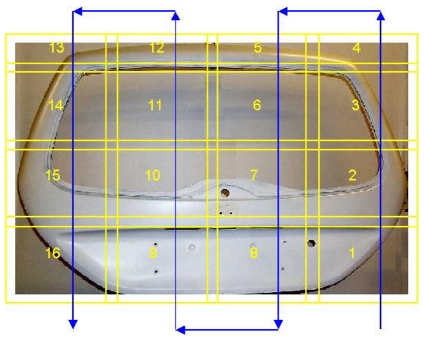
The car back door. The sequence planned for the view acquisition is overlapped.

An interesting application is the measurement of large, free-form objects, as that one shown in Figure 9. The complexity and the dimension of the target suggests the use of a 3D imaging sensor belonging to the class of stereo vision or structured light devices, and the acquisition of multiple point clouds, to cover the whole surface. In addition, to obtain measurement processes suitable for the industrial production field, the alignment of the views should be carried out automatically, keeping the operator intervention at minimum.

**Figure 10.**
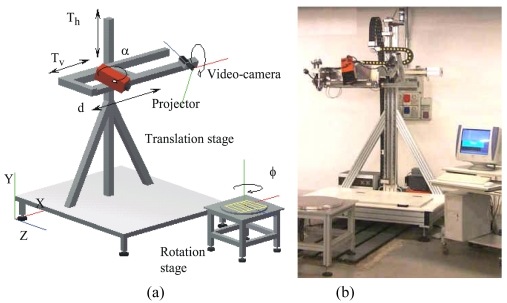
The 3D instrument developed at the laboratory. (a) Layout of the system. (b) Photograph of the device.

The stereo vision based Ortigo 200 system (Cognitens, Inc) and the Romer Omega mobile scan arm, equipped with the laser stripe R Scan (Hexagon, Inc) are two examples of market available instruments specifically developed for quality control. Our Laboratory has designed and developed the system shown in Figure 10 [82]. The system layout is shown in Figure 10a.

The camera-projector pair that is configured following the triangulation geometry shown in Figure 1 represents the optical head. The light coding approach is based on the well known Gray-Code and Phase-Shift (GCPS) projection technique [83]. The geometry of the optical head can be varied depending on the resolution required by the measurement. In detail, both the orientation and the distance of the projector with respect to the video-camera can be varied respectively within the rotation interval 20°–50° (α is the angle of rotation) and the translation interval 400 mm–1 m (d is the mutual distance between the optical devices). The optical head is mounted on a moving stage, allowing its translation along directions X and Y; in this way, areas up to 2 m^2^ can be scanned and larger surfaces can be measured by acquiring and aligning a number of separate patches. Moreover, objects presenting circular symmetry can be placed on a rotation stage: this enables the acquisition of patches at different values of the rotation angle, denoted by ϕ in the figure.

The system, shown in Figure 10b is equipped with stepped motors, to fully control the rotation and the translation of the optical head. The sequence of acquisition of the range images is decided on the basis of the dimension and the shape of the object. Objects characterized by predominant shapes along the X and the Y axes are acquired by moving the optical head horizontally and vertically, at steps depending on the field of view. In this case, only translation matrices are used to align the range images. In the general case of objects which can be completely measured by a combination of rotation and displacement of the optical head, the alignment is performed by using a combination of rotation and translation matrices.

**Figure 11.**
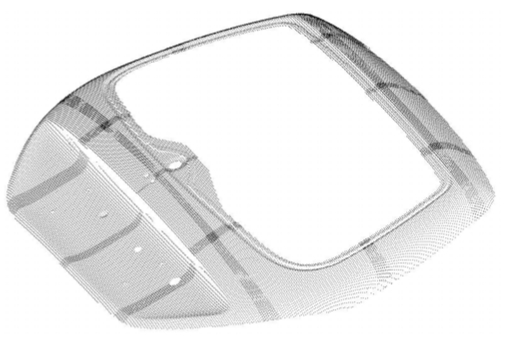
Whole point cloud of the car back door.

The measurement of the car back door (1.20 m height by 1.60 m width) required the acquisition of the patches labeled from 1 to 13 in Figure 9, (the blue line represents the acquisition path); each patch was 400 mm large and 300 mm high. The time to acquire a single patch was 4sec. The variability of the measurement is ±30 μm, with a resolution of 70 μm. The whole point cloud is shown in Figure 11. The overall error is within 0.1 mm, taking into account the contribution of the matching between adjacent views.

#### 3.1.3 Reverse engineering of free-form shapes

Reverse engineering is the process of creating a set of engineering specifications and drawings directly from the inspection of an object, a geometric model of which is obtained from sensor data. For objects with geometric regularity, it is customary to generate them analytically, using one of several geometric model schemes. For free-form shapes that do not present regular geometric properties, it is necessary to measure them and to create the 3D model for their 3D features representation.

**Figure 12.**
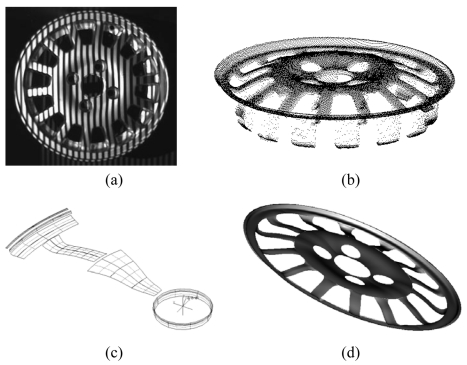
Process to create the CAD model of a free-form surface. (a) Optical acquisition of the wheel rim used as the target. (b) Range image of the top side of the wheel rim. (c) Generation of the paths for the digitization by using the CMM. (d) CAD model of the target.

The current industrial technology for precise measurement of 3-D object involves contact scanning by Coordinate Measuring Machines (CMMs). This technique is precise and widely used for the creation of the surface model [9]. As already mentioned, however, the primary disadvantage of this approach is the time wasting nature of the work. On the other hand, the use of 3D imaging sensors allows the collection of large amount of dimensional data in reasonably short time. However, the metrological performance of contactless sensors is usually lower than that of the CMM. The combination of optical and mechanical probes is strategic, in view of obtaining high quality of the CAD description at a dramatically reduced time. A survey of techniques developed in this field is in publications [84–87]. Our Laboratory, in collaboration with other partners, developed a novel methodology for the reverse engineering of free-form surfaces based on the integration of the measurement information from the system in Figure 10 and a CMM [88]. Figure 12 shows the basic steps of the method. The optical system rapidly gauges the shape of the wheel rim, as shown in Figure 12a. The acquired point cloud, shown in Figure 12b is the starting information for the reconstruction of a ‘rough’ CAD model of the object.

To this aim, we used a suitably developed program, called Visual Point, which allows the manipulation of the point clouds in the pre-processing of raw data. The CAD model was then elaborated by the PRO/Engineer software (PTC Inc). This model presented an accuracy of 0.5mm, which was sufficient to generate a collision-free inspection path on the CMM. In this way, the CMM could work independently from the operator. It acquired automatically a very dense point cloud with 4 μm of uncertainty, at dramatically reduced time. Figure 12c shows the partial reconstruction of the wheel rim after the first initial digitization. The measured points allowed us to obtain the accurate CAD model of the wheel rim shown in Figure 12d.

Reverse engineering processes can be accomplished even on a full-optical acquisition basis. In this case, 3D imaging sensors gauge the surfaces. This choice is optimal in Collaborative Design and Rapid Prototyping applications, which are typically involved in the design process of products that must have aesthetical appeal, such as the exterior of consumer products ranging from perfume bottles to automobiles. A considerable number of research projects have been carried out so far [89–92]. Among the projects developed at our Laboratory, we mention two examples.

**Figure 13.**
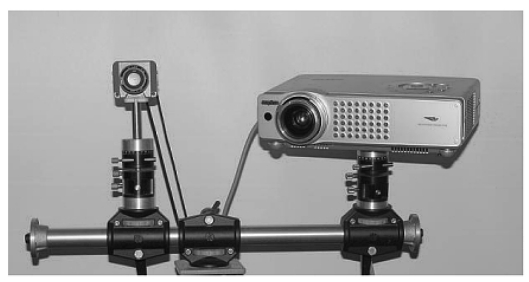
The low-cost whole-field structured light sensor designed for modeling applications.

**Figure 14.**
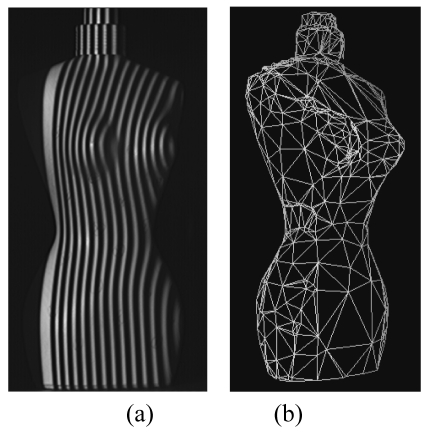
Example of the modeling of a perfume bottle. (a) Projection of the fringe pattern. (b) Triangle mesh that models the object shape.

The first is the development of the low-cost whole field structured light sensor shown in Figure 13. The optical head is composed by a market available, low-cost SVGA LCD projector, and the camera is a low-cost device at standard resolution. This sensor performs the measurement by projecting a single fringe pattern, similar to that shown in Figure 3c. The elaboration of the pattern is performed by means of a phase demodulation approach specifically designed to increase the measurement range [79]. The optical head is mounted on a bar, fixed on a robust tripod: special calibration allows us to adapt the system set-up dependently on the required input resolution and range. In contrast to the system shown in Figure 10, the full coverage of the shape is obtained by manually moving the instrument in the measurement scene, and by acquiring multiple point clouds. The procedure for their alignment exploits the correspondence between points in different adjacent views. The PolyWorks (InnovMetric Inc, Ca) suite of programs carries out this task. An application example is shown in Figure 14. The target object is a (135 × 60 × 45) mm^3^ (height × width × depth) perfume bottle, reproducing the form of a female body.

Figure 14a shows the projection of the fringe pattern during the measurement of a single point cloud. The acquisition of the target is accomplished by acquiring six views. These are aligned by using the ImAlign module of the PolyWorks suite. The accuracy of the 3D raw data is 233 μm, a reasonable value for the modeling process. This is carried out by using the ImMerge and the ImEdit modules of PolyWorks, for the creation and the optimization of the triangle mesh. The mesh is presented in Figure 14b.

**Figure 15.**
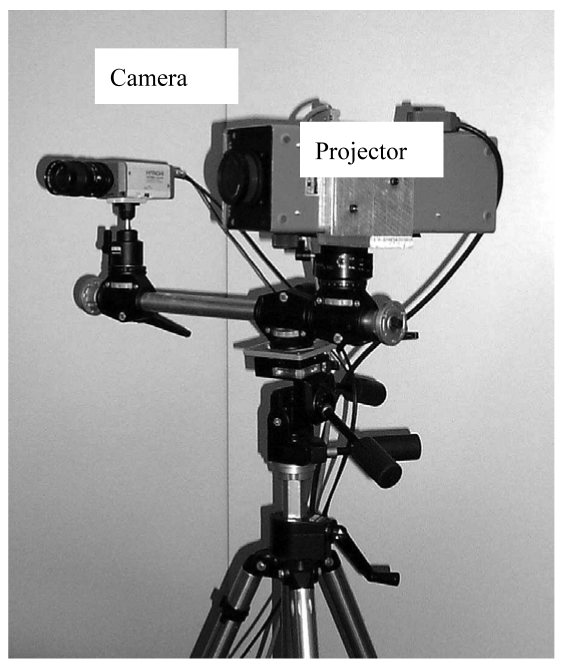
Photograph of OPL-3D.

The latter project is the development of the 3D imaging sensor shown in Figure 15. This system, called OPL-3D, is a whole-field structured light sensor that combines the GCPS coding procedure of the instrument in Figure 10 with a calibration approach similar to the one developed for the system in Figure 13 [25].

OPL-3D exhibits low-measurement uncertainty (120 μm) over large measurement areas (450 mm by 340 mm), linearly scalable in the case of smaller areas. It is portable and easy reconfigurable to be successfully used in different measurement conditions. It is now commercially available under the name of 3Dshape (OpenTechnologies s.r.l., Italy).

**Figure 16.**
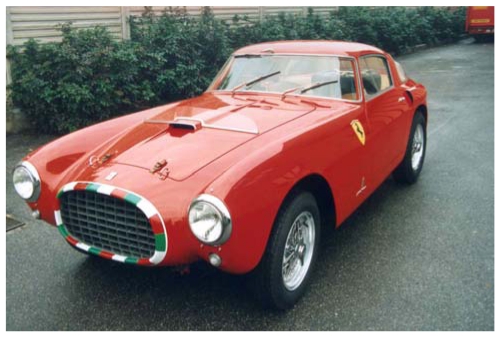
The Ferrari 250MM historical racing car.

**Figure 17.**
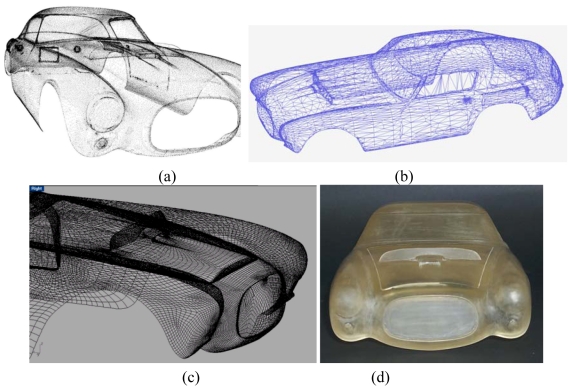
Example of full optical reverse engineering in the automotive domain by using OPL-3D. (a) Point cloud of the car body. (b) Triangle mesh. (c) CAD model. (d) Scaled prototype of the Ferrari.

This sensor is particularly suitable whenever the measurement problem requires high measurement performance and flexibility of reconfiguration. We fruitfully used it to carry out the complete acquisition of the car body of the Ferrari 250MM shown in Figure 16. The aim of this experiment was to assess the efficiency of the process from the acquisition to the modeling of the surfaces [93]. Figure 17 shows the performance of OPL-3D. The whole point cloud in Figure 17a was obtained by acquiring and merging 400 point clouds, at different resolution and dimension, depending on the details. The Geomagic suite of programs elaborated the 3D raw data. First, the triangle mesh was created (Figure 17b); then the CAD model was calculated starting from the mesh. The CAD model is shown in Figure 17c. The model was then exported in the STL format and input to a Rapid Prototyping machine, for the production of the 1:8 scaled copy, shown in Figure 17d.

### 3.2 Cultural heritage applications

**Figure 18.**
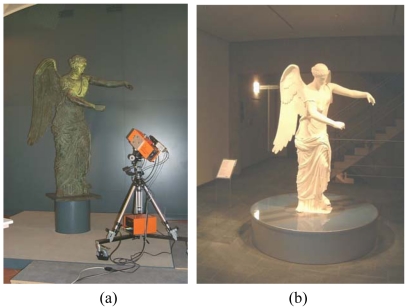
The Winged Victory of Brescia. (a) The original statue. (b) The full scale copy of the statue.

In the last years, the use of 3D imaging sensors for the contactless acquisition of cultural heritage has gained increased attention by museums and by the archaeological specialists [100]. A number of reasearch laboratories have worked on the modeling of statues [95–97], the 3D documentation and virtualizing of archaeological sites [98–100], the automated classification of pieces [101], and visualization applications [102]. The measurement and elaboration chain is still a reverse engineering process, similar to the one developed for the industrial field. Even the objectives are the same: acquisition, modeling and visualization are required in the cultural frame, to monitor the archaeological founds. In addition, the availability of 3D models opens the door to the virtual reproduction, for didactical and visualization purposes. Physical copies of the originals are produced by using Rapid Prototyping techniques: an example is the reproduction at full scale of the two meter high Winged Victory of Brescia, shown in Figure 18. Our Laboratory at the City Museum accomplished the acquisition in 2001 [103, 104]. The optical sensor was the OPL-3D.

**Figure 19.**
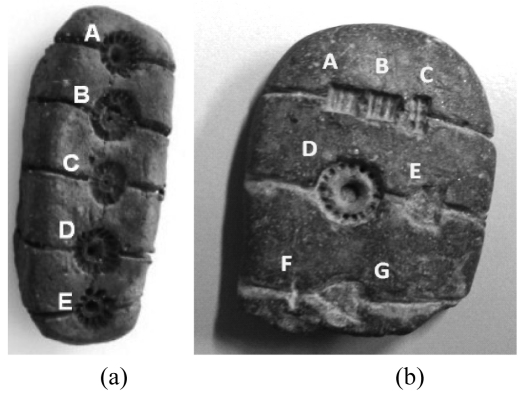
Examples of the ‘Tavolette enigmatiche’. (a) The ‘VESELE’ piece, found in Slovenia. (b) The LOVERE piece, found in Northern Italy.

One of the fields where 3D accurate acquisition is strategic is the documentation of finds. Traditional techniques require the use of contact probes and the hand-made drawing of the originals: this practice introduces (i) inaccuracies of the reproduction, (ii) loss of information, and (iii) subjectivity of the description. The most recent project we are working at focuses on the documentation of the so-called ‘Tavolette enigmatiche’, two examples of which are presented in Figure 19.

The ‘Tavolette enigmatiche’ are small baked-clay objects of prevalently ovoid shape of the 2,100-1,400 B.C. periods, engraved with symbols and drilled holes whose meaning is still unknown. Their presence in Italy and in many countries in the northeast Europe represents an ‘enigma’ for the specialists, since the shape and the orientation of the signs is very similar among the objects, even if they have been found in different geographical sites. Hence, we have been required to accomplish an extensive measurement campaign in Europe, and to produce and collect the 3D models in a unique database of 3D models, in view of their future study by the specialists. To carry out the measurement, we used the Vivid 910 sensor (Konica Minolta Inc.). Besides the measurement performances, that well suit to the resolutions required in this application, the system is rugged, portable, and fast in the setup and the acquisition processes. So far, 30 specimens have been acquired, modeled and organized in a database.

**Figure 20.**
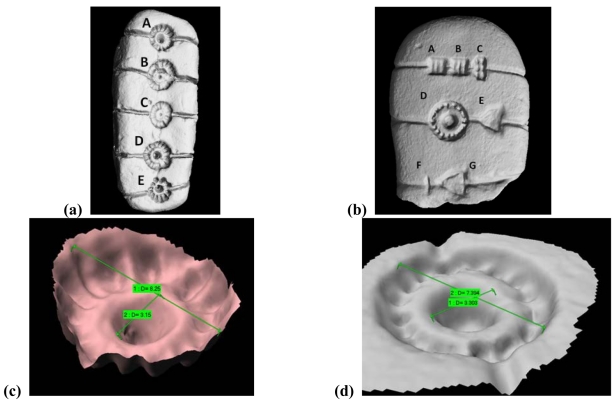
Modeling of the ‘Tavolette enigmatiche’. (a) 3D mesh of the ‘VESELE’ specimen. (b) 3D mesh of the ‘LOVERE’ specimen. (c) Zoom of the detail labeled by letter ‘A’ in the 3D model of Figure 19a. (d) Zoom of the detail labeled by letter ‘D’ in the 3D model of Figure 19b.

**Figure 21.**
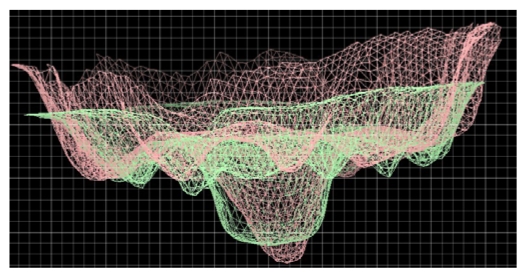
Superposition of the signs in Figure 20c and Figure 20d, to quantitatively evaluate their similarities.

Figure 20 shows the 3D models of the objects in Figure 19. Their resolution is 120 μm. The snapshots in Figure 20c and in Figure 20d are the zoomed views of the signs denoted by letter ‘A’ in the model of Figure 20a, and by the letter ‘D’ in the model in Figure 20b. The availability of these representations is of outmost importance in view of carrying out the comparative analysis among the objects on a quantitative, objective basis. Figure 21 shows an example: the two signs are overlapped in the same reference frame, and can be compared. The work is still in progress: we are now developing the algorithms dedicated at automatically retrieving and correlating similar symbols among different founds [105].

### 3.3 Medical applications

3D imaging sensors designed specifically for the living human body are becoming increasingly available, providing high-resolution surface data with capability to build numerically and perceptually accurate digital body models [106–108]. 3D data are used in prosthetics design, plastic surgery, orthopaedy, prothetic orthodontics, surgery and dermatology [16, 109]. An intense research activity focuses onto the construction of dynamic 3D digital models for the study of pose and walking, and for real time surgical applications [110, 111]. Very recently, uses of 3D imaging sensors have been proposed in forensic medicine, even in combination with radiological data, for the complete documentation of injuries [112–116].

**Figure 22.**
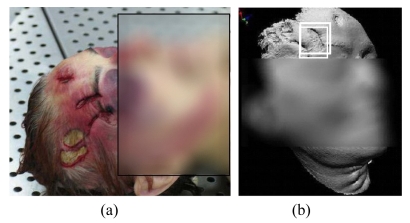

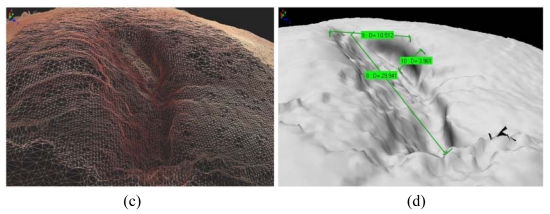
Example of post mortem analysis. (a) The victim. (b) 3D model of the face. (c) Zoom of the framed lesion (color wireframe). (d) Example of the measurements (the measurement unit is mm).

A research activity dealing with the *post mortem* analysis of lesions on soft tissues and bones was developed at our Laboratory, in collaboration with the Institute of Legal Medicine of Milan. The aim was to study the feasibility of using 3D imaging sensors in order to assess a unique, precise and reliable method of investigation, providing accurate information about the origin of lesions and the modality of the event [117, 118]. In this project, we used the Vivid 910 system to perform the measurements during the autopsy. One example of the tests performed so far is shown in Figure 22.

This case refers to a woman killed by a blow with a metal pipe. The face of the victim, shown in Figure 22a, presented numerous lacerated and contused lesions. The optical acquisition resulted into the 3D model in Figure 22b (the elaboration was performed in the PolyWorks software environment). This model presents a high degree of fidelity with respect to the original surfaces, since the 3D raw data showed a resolution of 150 μm. Figure 22c shows the wireframe representation of the lesion framed in Figure 22b, and highlights the quality of the representation.

In Figure 22d, an example of the measurements that can be carried out is presented. The 3D model represents a quantitative, objective database of measurements, which different operators can share for subsequent analysis. The sensor proved to be adequate to this application, both in terms of the measurement quality and of the speed of acquisition.

### 3.4 Crime scene documentation

Crime scene investigation represents one of the fields where the use of contactless, accurate, fast acquisition systems would be of great advantage. This activity indeed requires the complete, accurate and objective description of a very complex and intrinsically three-dimensional scene, which should be documented at minimum invasiveness and time.

Traditional techniques, based on the use of contact rulers and on the two-dimensional acquisition of the scenes, do not match all these requirements: hence, in recent times, investigation techniques have moved toward more advanced methods, such as photograph stitching, virtual reality and photogrammetry [113]. These methods have partially fulfilled the lacks of the traditional approaches. However, neither they intrinsically produce three-dimensional information (as in the case of stitching) nor they provide full contactless measurement (as in the case of photogrammetry).

3D imaging sensors show promising to overcome the previously described limitations. Previous research was aimed at assessing the feasibility of using laser structured light sustems and video pairs for the documentation and 3D rendering of crime scenes [119, 120] The activity developed at our Laboratory in this field began in 2004, in collaboration with the UACV (Violent Crime Analysis Unit), a special unit of the Italian State Police.

The main goal was to assess the feasibility of using long-range three-dimensional sensors for the accurate, digital storing of crime scenes. In this research, the commercial HDS3000 laser scanner was used for data acquisition. This device is a Time-of-Flight system, originally developed for geology and civil engineering applications. It allows the 3D gauging of the surfaces up to 15 m, with resolution of 20 mm, and the acquisition up to 2 m with resolution of 2 mm.

**Figure 23.**
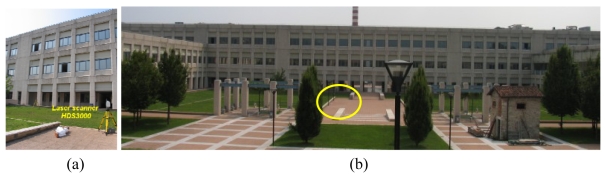
The simulated crime scene.

**Figure 24.**
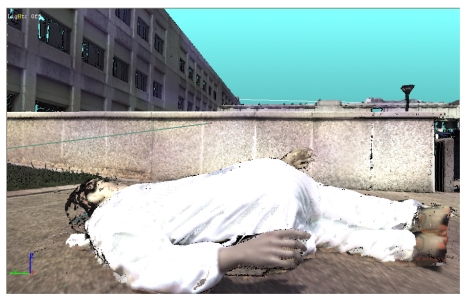
3D model of the victim area.

The system was used to acquire a simulated crime scene. The victim was simulated by using the dummy shown in Figure 23a. The photograph in Figure 23b presents the whole scene: the area in Figure 23a in highlighted by the oval. The ‘victim’ area was acquired at the highest resolution, and the 3D model shown in Figure 24 was created.

**Figure 25.**
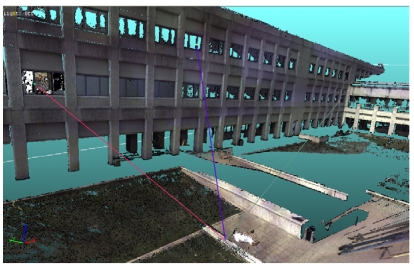
3D model of the crime scene.

The surroundings were captured by using the sensor at the lowest resolution, over a 15 meters long range. The resulting 3D model was then merged to the one corresponding to the victim, and the scene was modeled as in Figure 25. The combination of the models in a multi-resolution approach was strategic to maintain the level of details and, at the same time, to reduce the data amount. The availability of this whole model turned out to be crucial for subsequent analysis and visualization. The model is metrically traceable, and a large variety of measurement could be accomplished. Some examples are the measurement of areas, of sections, and of distances among the details of the scene. In the figure, one of the elaborations allowed by this representation is shown: it deals with the study of the possible directions of projectiles [121].

## 4. Conclusions

In this paper, a review of the most important techniques and sensors for the optical 3D measurement of surfaces has been presented. The aim was to highlight the wide range of measurement problems that can be solved by using 3D imaging sensors. A number of examples have been shown, with reference to the experience carried out at our Laboratory in the last ten years.

The overview of the state-of-art on 3D acquisition systems proposed in this paper yields a number of conclusive remarks. The first concerns the cost of the equipment for 3D acquisition. Most of the equipment available is still remarkably expensive, and this still represents an obstacle to a much wider distribution of 3D systems especially inside SMEs. However, we assist to a trend towards a decrease in costs, mainly due to (i) the increase in the number of manufacturers, and hence an increased competition, and (ii) the technology evolution, that offers optical/electronic components at low costs.

The second remark concerns the fact that, for most of the applications of real relevance in industry, the use of 3D acquisition is by no means a trivial task: the systems are still rather complex to use, and skilled personnel is required to operate them. Here again, future trends may consist in an overall simplification of the acquisition procedures, to make the system usage affordable for semi-skilled SME operators.

A third remark, which is crucial from the metrologist's viewpoint, is the need of norms to guarantee the traceability of 3D measurements to recognized standards. Being the 3D acquisition rather new, these norms are not yet mature.

A final remark concerns the fact that 3D systems, in many complex metrological issues, may not represent the solution of the problem when used alone; the concurrent use of combinations of contact and non-contact systems, including 3D systems, may be required for a full metrological solution.

Given the above remarks, the future of 3D systems is indeed promising, since the applications of 3D acquisition are dramatically increasing: 3D imaging is now a fundamental part of automation, robotics, of Rapid Prototyping, and 3D modeling. It is also easy to predict that, if the future of 3D rendering through holography will be as positive as it is expected, the applications will expand even more.

## Figures and Tables

**Table 1.** Classification of 3D imaging techniques.

	Triangulation	Time delay	Monocular Images	Passive	Active	Direct	Indirect	Range	Surface Orientation
Laser triangulators	X				X	X		X	
Structured light	X				X	X		X	
Stereo vision	X			X		X		X	
Photogrammetry	X			X		X		X	
Time of Flight		X			X	X		X	
Interferometry		X			X	X		X	
Moiré fringe range contours			X		X		X	X	
Shape from focusing			X	X	X		X	X	
Shape from shadows			X		X		X	X	
Texture gradients			X	X			X		X
Shape from shading			X		X		X		X
Shape from photometry			X		X		X		X

**Table 2.** Comparison of optical range imaging techniques.

**TECHNOLOGY**	**STRENGTH**	**WEAKNESS**
Laser triangulators	Relative simplicity Performance generally independent of ambient light High data acquisition rate	Safety constraint associated with the use of laser source Limited range and measurement volume Missing data in correspondence with occlusions and shadows Cost
Structured Light	High data acquisition rate Intermediate measurement volume Performance generally dependent of ambient light	Safety constraints, if laser based Computationally middle-complex Missing data in correspondence with occlusions and shadows Cost
Stereo Vision	Simple and inexpensive High accuracy on well-defined targets	Computation demanding Sparse data covering Limited to well defined scenes Low data acquisition rate
Photogrammetry	Simple and inexpensive High accuracy on well-defined targets	Computation demanding Sparse data covering Limited to well defined scenes Low data acquisition rate
Time-of-Flight	Medium to large measurement range Good data acquisition rate Performance generally independent of ambient light	Cost Accuracy is inferior to triangulation at close ranges
Interferometry	Sub-micron accuracy in micro-ranges	Measurement capability limited to quasi-flat surfaces Cost Limited applicability in industrial environment
Moiré fringe range contours	Simple and low cost Short ranges	Limited to the measurement of smooth surfaces
Shape from focusing	Simple and inexpensive Available sensors for surface inspection and microprofilometry	Limited fields of view Non-uniform spatial resolution Performance affected by ambient light (if passive)
Shape from shadows	Low cost Limited demand for computing power	Low accuracy
Texture gradients	Simple and low cost	Low accuracy
Shape from shading	Simple and low cost	Low accuracy
